# Praziquantel is an effective drug for the treatment of *Schistosoma Mansoni* infection among school-aged children in Northwest Ethiopia

**DOI:** 10.1186/s41182-020-00204-z

**Published:** 2020-04-29

**Authors:** Addisu Tesfie, Gebeyaw Getnet, Aberham Abere, Gebeyehu Yihenew, Yerega Belete, Melkayehu Kassa, Addisu Gize

**Affiliations:** 1 Amhara Public Health Institute-Dessie Branch, Amhara Region, Ethiopia; 2 School of Biomedical Science, College of Medicine and Health Science, University of Gondar, Gondar, Ethiopia; 3 Department of Microbiology, St. Paul’s Hospital Millennium Medical College, Addis Ababa, Ethiopia

**Keywords:** Praziquantel, Schistosomiasis, Effective, Ethiopia

## Abstract

**Background:**

The appropriate drug for the treatment of schistosomiasis is praziquantel. However, low cure rate and existence of drug resistance both in vivo and in vitro were reported in different endemic areas. Hence, the aim of this study was to evaluate the effectiveness of praziquantel for *Schistosoma mansoni* (*S. mansoni*) treatment.

**Methods:**

A cross-sectional study was conducted in Sanja General Primary School, North Gondar Zone, Amhara region, Northwest of Ethiopia, from March to April, 2017. A total of 245 participants were selected using systematic random sampling. A stool specimen was collected from each participant and examined for *S. mansoni* ova load count using Kato–Katz technique. Two hundred four infected participants were treated with a single oral dose of praziquantel 40 mg/kg. Four weeks later post-treatment, stool specimens were collected from 176 study participants. The samples were collected using similar procedures like the pre-treatment phase to see egg reduction and cure status. Data were entered and analyzed using SPSS version 20.0 Pearson chi-square (*χ*^2^) was used to determine the association of effectiveness of the drug with the average egg count, age group, and sex. *P* value ≤ 0.05 at 95% CI was considered statistically significant.

**Results:**

Pre-treatment prevalence of *S. mansoni* infection was 83.3% (204/245) with geometric mean egg count of 357.8. In those not cured post-treatment, the prevalence and egg per gram in geometric mean egg count were 13.1% and 77.6 respectively.

After 4 weeks of administration of praziquantel, the cure rate was 86.9% with egg reduction rate of 78.3%. Effectiveness of the drug was not statistically associated with sex, age group, and pre-treatment intensity of infection.

**Conclusion:**

*S. mansoni* prevalence was high. Praziquantel is an effective drug for the treatment of *S. mansoni*. This high prevalence of *S. mansoni* requires mass drug administration of praziquantel.

## Introduction

Schistosomiasis is a tropical parasitic disease and a major public health concern in developing countries where sanitation and access for safe drinking water are limited [1, 2].

Worldwide, the causative agents for the human disease are *S. haematobium*, *S. mansoni*, and *S. japonicum*, while *S. mekongi* and *S. intercalatum* are restricted in certain geographical locations. The first two, *S. haematobium* and *S. mansoni* cause intestinal and urinary diseases respectively, they are also called the African schistosomiasis [1, 3]. More than 230 million people are infected with schistosomiasis [4], out of them over 90% live in Sub-Saharan Africa [5]. It is also estimated that 779 million people are at risk of acquiring the disease; again majority of population at risk is from Sub-Saharan Africa [1, 6]. Next to malaria, the most critical neglected tropical diseases in the developing countries are found in 48 African countries [6].

In Ethiopia, the two species of schistosoma, *S. mansoni* and *S. haematobium*, are public health concern, especially *S. mansoni* which is widely spread throughout the nation [7]. Schistosomiasis is common in northern region as compared to others regions of Ethiopia [8]. Different studies of *S. mansoni* among school children showed variable prevalence: 67% in Northwest Ethiopia [7], 5.95% in Tigray [9], 67.6% in Finchaa valley [10, 11], and recent study done among Sanja Primary school children showed prevalence of 84.6% which was identified as new foci of transmission [12].

Pathophysiology of schistosomiasis is associated with immunologic reactions of body to schistosoma eggs trapped in tissues. Antigens released from egg stimulate a granulomatous reaction involving T cells, macrophages and eosinophils that results in clinical disease. Symptoms and signs depend on the number and location of eggs trapped in tissues. Initially, the inflammatory reaction is readily reversible. In latter stages of the disease, the pathology is associated with collagen deposition and fibrosis, resulting in organ damage that may be only partially reversible [13].

Impaired cognitive potential, hepatosplenomegaly, anemia, urinary bladder cancer, and stunted growth are devastating effects resulting from schistosomiasis [14]. Diseased individuals manifest clinically with granuloma and fibrosis in the intestine or liver leading to other complications including hemorrhage. In chronically infected individuals, serious complications are imminent, such as renal disorders due to the immune complex or exposure to secondary bacterial or viral infection [2–7].

For this study, praziquantel (PZQ) is the appropriate drug of choice due to its safety, low cost, patient compliance, and effective treatment for all five schistosome species [15]. It is a broad spectrum schistosomacide of pyrazinoisoquinoline derivative, which is stable under normal conditions and soluble in organic solvents [16]. Praziquantel affects the permeability of the cell membrane resulting in the contraction of schistosome by increasing calcium influx. The drug further causes vacuolization and disintegration of the schistosome tegument. The effect is more marked on adult worms compared to young worms. Secondary effects are inhibition of glucose uptake, lowering of glycogen levels, and stimulation of lactate release [13]. Currently, the drug is administered at the standard single oral dose of 40 mg/kg body weight and is the mainstay drug recommended by WHO for chemo-preventive therapy [5, 17]. Even though the drug has been reported as effective, safe, and generally without serious side effects, low cure rates were reported from studies in Senegal and Egypt [18, 19]. It has also been reported that praziquantel can cause poor coordination, abdominal pain, vomiting, headache, and allergic reaction side effects [20]. There are limited number of studies on *S. mansoni* PZQ treatment [2, 10] in addition to reports from infected patients, while taking appropriate doses of PZQ’s inability to cure. This also raises the question of whether there is a resistant to the current available drug. However, egg reduction rate (ERR) was still over 80%. The most likely explanations for this low cure rate were the density of infection at the beginning, high transmission, and immunodeficiency, though the possibilities of drug resistance or tolerance are not investigated [21]. In Ethiopia, it is not well investigated on the effectiveness of PZQ against S. mansoni species though few studies have been done in other transmission foci. Hence, a study on treatment effectiveness is very crucial at this point in time, as the National Strategic Plan for control of schistosomiasis is under development. It will also be followed by mass drug administration based on the countrywide mapping result. Consequently, this study aimed to determine the effect of PZQ against *S. mansoni* in school-aged children, Sanja Town, Tacharmachiho district, Gondar, Northwest Ethiopia.

## Materials and methods

### Study area

The study was conducted among students of Sanja General Primary School, Sanja town, Tacharmachiho district, North Gondar, Amhara region, Northwest of Ethiopia, from March to April 2017. The study area is found 792 km away from Addis Ababa, North Gondar Zone, Amhara region in the Northwest of Ethiopia. The weather is hot and reaches 29 to 31 °C. The area is crossed by Sanja River and Maho stream which are the main sources of *S. mansoni* infection. Sanja River is big and used for lots of services for the community living there. Maho is a small stream which flows slowly and stops flowing usually by February until the rainy season returns back in June. According to 2007 census, the total population was approximately 7255, out of which 3591 were males and 3664 were females. The school is located on the west side of the main road and had 2079 students from grade 1 to 8, of which 872 were males and 1207 were females [12].

### Study design

A cross-sectional study was conducted among children infected by *S. mansoni*; they were assessed both at the baseline and 4 weeks later post-treatment by Praziquantel. Stool was taken from every study subject for examination by Kato-Katz technique.

### Source population

The source population was all children attending their education at Sanja General Elementary School with in the specified academic year of 2017.

### Study population

These are the school children who satisfied inclusion criteria and enrolled at the school during the study period.

### Inclusion and exclusion criteria

School children aged ≥ 6 years, with no recent anthelmintic treatment (within the last 6 month), no involvement in any other clinical trial, and children who were willing to participate were included.

Children, who vomited within 4 h after drug administration, took anti-worm drugs within the past 6 months, and those who submitted diarrheal stool specimens were excluded from the study.

### Operational definition

Cure rate: no detection of ova of *S. mansoni* in stool sample after treatment among those who were excreting ova of the parasite. It is also seen the percentage of children who were positive before the study and those who were going to egg free by the Kato–Katz technique after 4 weeks of medication.

Egg reduction: the decrease in the level of infection was checked indirectly using the egg count calculating geometric egg count using the following formula [22].

$$ \mathrm{Egg}\ \mathrm{reducation}\ \mathrm{rate}=\left(1-\frac{\mathrm{geometric}\ \mathrm{mean}\ \mathrm{of}\ \mathrm{egg}\ \mathrm{per}\ \mathrm{gram}\ \mathrm{after}\ \mathrm{treatment}}{\mathrm{geometric}\ \mathrm{mean}\ \mathrm{of}\ \mathrm{egg}\ \mathrm{per}\ \mathrm{gram}\ \mathrm{before}\ \mathrm{treatment}}\right)\times 100 $$

### Sample size and sampling technique

Sample size was determined by applying the single population proportion, *n* = (*Z*α/2) 2 × *p* (1−*p*)/*w*2), Where, *n* = sample size, *z* = z statistic for a level of confidence, *w* = precision, and *p* = expected prevalence or proportional 95% level of confidence with a margin of error of 5% and 79.5% prevalence from previous result taken in the study area which resulted in the 251 samples. Since the target population is *N* = 2079 from the school record used as a sampling frame is < 10,000, considering the correction formula (*n*/1 + *n*/*N*), where *n* is 251 and *N* is 2079, we obtained 224. By adding a 10% non-response rate, we included 245 students in this study. The calculated sample size was high compared to the WHO guideline [23] of minimum sample of students to be screened and this was done intentionally to get more positive cases.

### Sampling technique

Probability sampling method was used to select the required study participants. Based on their level of education, students were stratified (grade 1 to 8). After stratification, sample was proportionally allocated for each grade according to the number of students. Finally, selection was done using systematic random sampling technique via lottery method. Whenever the selected student was absent, student before or after the enrolled participant was selected for replacement.

### Data collection and laboratory methods

Using clean, screw caped, and labeled plastic containers 2 g of stool was collected from the study subjects. Socio-demographic data were collected by trained nurse. Stool samples were processed for microscopic examination using 41.7 mg Kato–Katz template. Kato–Katz thick smears were prepared in Sanja town and the samples were sent to the University of Gondar laboratory for microscopic examination. Snacks were given for children who were positive for the parasite before treatment by a single standard dose of 40 mg/kg praziquantel. Drugs were administered by trained nurses and the children were followed for 4 h for adverse effects and any complication. After 4 weeks of post-treatment, samples were collected and examined in the laboratory based on the standard procedure to evaluate treatment effectiveness.

### Treatment and effectiveness evaluation

The evaluated drug was obtained from Ethiopian Red Cross pharmacy, Gondar branch, Northwest Ethiopia. Treatment effectiveness was determined by means of cure rate (CR, percentage of children positive at the pre-treatment baseline who will become egg-negative 4 weeks after treatment, and egg reduction rate (ERR, reduction in the group’s geometric mean fecal egg count comparing the before and after treatment) [23].

### Quality assurance

Collection and examination of the specimens were based on the standard operating procedure (SOP) for the Kato–Katz technique. Proper specimens labeling and matching with respective identification numbers were checked. Training was given for data collectors who were nurse and laboratory technicians. The evaluated drug was certified by national drug control authority (with stamp). For quality control, 10% of the slides were randomly selected and re-examined by experienced medical laboratory technician at the University of Gondar Parasitology laboratory. There were no discrepancies observed between the original egg counts and by the experienced laboratory technician. Data were double entered to minimize errors.

### Data analysis and interpretation

Data were entered and analyzed using the SPSS version 20.0 statistical software package. Different variables were described and characterized by frequency distribution. The prevalence of schistosomiasis was computed as percentage. Eggs per gram of stool (EPG) were obtained by multiplying the number of eggs per Kato–Katz slides by 24, and the intensity of *S. mansoni* infection was expressed as geometric mean egg count. Classes of intensity were defined as light (1–99 EPG), moderate (100–399 EPG), and heavy (≥ 400 EPG) [1]. Cure rate was calculated as the proportion of individuals found negative for *S. mansoni* eggs among those examined 4 weeks after treatment. Egg reduction rate was calculated as 1-(EPG (geometric mean) after treatment /EPG before treatment) × 100. Pearson chi-square (*χ*^2^) was used to determine the association of effectiveness of praziquantel with the mean egg count, age, and sex. *P* value ≤ 0.05 at 95% CI was considered statistically significant.

### Ethical considerations

Ethical approval was obtained from the school of Biomedical and laboratory science (SBLS), and permission from Tacharmachiho district Health and Education offices, and Sanja General Elementary School director.

Written informed consent also obtained from legal guardians or parents of participating children after clearly explaining the objective of the study. Assent form was also taken from adolescent groups. Parents/guardians were told as each participant is free to withdraw consent and discontinue participation in this study at any time. Children who tested positive for *S. mansoni* both before and after treatment were treated with praziquantel at a single dose of 40 mg/kg body weight and those who tested positive for soil-transmitted helminths were also treated with a standard regimen of anthelmintic (with 400 mg albendazole). The individual results of any investigation were remained confidential.

## Results

### Pre- and post-treatment prevalence and intensity of infection

A total of 245 school children (163 males and 82 females) participated in this study; their mean age was 10.1 years (age range 6–18 years) with the highest proportion (56.3%) was in the age group 10–14. The prevalence of *S. mansoni* infection before treatment was 83.3% (204/245). Of these, 26.5%, 37.3%, 36.3% had light, moderate, and heavy intensity of infection, respectively. The prevalence dropped to 10.8% (23/176) 4 weeks post-treatment with 88.7% reduction in prevalence (Table 1, Table 2, Table 3).

**Table 1:** Pre and post-treatment prevalence (%) and intensity (EPG) of *S. mansoni* infection among Sanja General Elementary school children, Northwest Ethiopia, 2017

Variables	Pre-treatment			Post-treatment		
	% positive (no. pos./no exam)	GMEPG	% positive (no. pos./no exam)	GMEPG	% reduction in prevalence	% reduction in GMEPG
Age groups						
6–9 years	80.9 (85/105)	421.8	13.8 (11/80)	77.79	87.1	81.4
10–14 years	85.5 (118/138)	303.34	11.6 (11/95)	74.3	90.7	70.8
≥ 15 years	50 (1/2)	360	100 (1)	120	0	66.7
*χ*^2^ (*p*)	2.89 (0.193)		4.59 (0.17)	17.3 (0.33)		
**Sex**						
Male	83.1 (138/166)	415.06	12.1(14/116)	102	89.6	75.4
Female	83.5 (66/79)	284.03	15 (9/60)	51	86.4	80.1
*χ*^2^ (*p*)	0.007 (0.936)		5.92 (0.67)	5.92 (0.67)		

*χ*^2^ chi-square, *p p* value, *no* number of, *GMEPG* geometric mean egg per gram

Four weeks post-treatment, stool samples were collected from 176 children who had tested positive and been treated at the baseline survey (Table 2). Of these, 23 were still positive for *S. mansoni ova*; 54.5% and 45.5 % had light and moderate intensity of infections, respectively. The lowest prevalence after treatment was 11.6% among 10–14 years old children. Both prevalence and intensity of infection were not significantly associated with age and sex of study participants during pre-treatment as well as post-treatment (Table 3).

**Table 2:** Pre-treatment prevalence (%) and proportion of classes of *S. mansoni* infection by age group and sex in Sanja General Elementary School children, Sanja town, Tacharmachiho district, Northwest Ethiopia, from March to April 2017

Variable	Pre-treatment classes of intensity						
Age group (years)	No. exam	No. pos.	% prevalence	% light (*n*)	% moderate (*n*)	% heavy (*n*)	*χ*^2^ (*p*)
6–9	105	85	80.9	29.4 (25)	31.8 (27)	38.8 (33)	3.38 (0.47)
10–14	138	118	85.5	24.6 (29)	40.7 (48)	34.7 (41)	
≥ 15	2	1	50	0	100 (1)	0	
Sex							
Male	166	138	83.1	27.5(38)	34.1 (47)	38.4 (53)	1.89 (0.39)
Female	79	66	83.5	24.2 (16)	49.9 (29)	31.8 (21)	
Total	245	204	83.3	26.5 (54)	37.3 (76)	36.3 (74)	

*χ*^2^ chi-square, *p p* value, *No* number, *Pos* positive

**Table 3:** Post-treatment prevalence (%) and proportion of classes of *S. mansoni* infection by age group and sex in Sanja General Elementary School children, Sanja town, Tacharmachiho district, Northwest Ethiopia, from March to April 2017

Variable	No. exam	No. pos	Post-treatment classes of intensity			
			% prevalence	% light (*n*)	Moderate % (*n*)	*χ*^2^ (*p*)
Age groups						
6–9 years	80	11	13.8	54.5(6)	45.5(5)	3.29(0.60)
10–14 years	95	11	11.6	72.7(8)	27.3(3)	
**Sex**						
Males	116	14	12.1	50(7)	50(7)	1.86(0.45)
Females	60	9	15	77.8(7)	22.2(2)	
Total	**176**	**23**	**13.1**	**60.9** (**14**)	**39.1** (**9**)	

*χ*^2^ chi-square, *p p* value, *No* number, *Pos* positive

### Egg reduction and cure rates

The overall pre-treatment intensity of infection was 357.8 GMEPG, which fell to 78 GMEPG 4 weeks post-treatment among those who were not cured. Hence, the overall ERR among those who were not cured was 78.3%, 75.4% among boys and 80.1% among girls. The egg reduction rate across the age groups was 81.4%, 70.8%, and 66.7% among children aged 9 years and younger, among 10–14 age group, and among those 15 years and older respectively. Intensity of infection after treatment and overall ERR was not significantly associated with either sex or age of the study participants. However, there were two exceptional cases in which mean egg count was increased after treatment (with ERR of − 17.5%).

The overall CR of *S. mansoni* infected individuals was 86.9% 4 weeks post-treatment, 87.9% among boys and 85% among girls. Within the age groups, the CR was 86.3% among children aged 9 years and younger, 88.4% in the 10–14 age groups and 0% among those 15 years and older. Both CR rate and ERR were not statistically associated with age, sex, and pre-treatment classes of intensity of infection.

## Discussion

This study found that the total pre-treatment intensity of infection to be357.8 EPG. Four weeks post-treatment with a single oral dose of 40 mg/kg body weight praziquantel, the intensity was reduced to 77.5 eggs and yielded an overall egg reduction rate of 78.3% among those not cured. This was in line with a study done in Western Côte d'Ivoire 79.9% reduction [18], but this finding was higher than the finding of a study conducted in the rural community of Côte d'Ivoire which was (61.4%), in Egypt (71.2%) [24], in Wondo Genet, Southern Ethiopia (68.2%) [25]. But lower than Mwea division central province, Kenya (92.6%) [26], Fincha valley, Western Ethiopia (99.5%) [10].

This study had also revealed an overall CR of 83.3%. This is concordant to a study conducted elsewhere in Ethiopia with cure rate of 82.89% [10]. However, the result was higher than the finding that was reported from CotdeIvore (71.6%) [18], and 60.9% in rural community of CotdeIvore, 74.9% in Wondo Genet, Southern Ethiopia [25]. Conversely, the cure rate in this study was lower than those findings that are reported by the studies conducted elsewhere in Ethiopia, 94% in northwest Ethiopia [27]. The variation of this study findings with that of the previous study findings with in Ethiopia might be due to sample size difference.

The difference in brands of the drug used might be the cause for variation on ERR and CR results. There was a study conducted on the impact of drug brand and dosage on the bioavailability and efficacy of PZQ in Egypt, among profoundly infected cases that were treated with PZQ; EPICO pharmaceuticals, Cairo, Egypt produced lower efficacy as compared to PZQ of brand Biltricide; Bayer AG, Leverkusen, Germany. Poor bioavailability of different drug brands due to genetic or dietary factors might result different efficacy outcomes [28].

The other possible reason for variable effectiveness might also be due to difference in time gap used to evaluate the effectiveness results after drug administration in which some used the time interval of 6 weeks others 4 weeks. Parasitological cure rate might be underestimated in those areas where individuals have a probability of being infected with either adult schistosomes or the juvenile stages of the schistosome. Largely, the younger parasites may develop into egg-laying adults due to their insensitivity to praziquantel [28]. In such case, the sufficient time for drug effectiveness needs to be evaluated. The evaluation of effectiveness in this study was done after 28 days following praziquantel administration. This is in line with findings of some of previous studies that were conducted in different study areas.

Moreover, variable effectiveness might be attributable to differences related to the host and parasite [28]. The high levels of parasite transmission and the recent nature of the host might be considered as a main set of factors. Most infected subjects harbor intensively high worm loads and are exposed constantly to new infections in the areas with high prevalence and intensity of infection. Many individuals have possibility to carry pre-patent infections that are not susceptible to praziquantel. They also have a possibility to be re-infected rapidly after treatment. The above two conditions could be a reason for observed low CR in this study. The new infections, even shortly after treatment during follow-up, may be diagnosed and identified as treatment failures. The detection of a huge number of immature stages of schistosome in different study situations also affect results of CR and ERR. Consequently, it is possible to presume that praziquantel still kills most schistosome worms, but not all, in high transmission foci. PZQ kills the worms at a normal rate in the societies with high worm burden. This could be mistaken as malfunction of treatment because the amount of worms enduring the treatment is enough to preserve positive to infection. The egg counts might be lower in most subjects [1, 28].

The failure of treatment is related to variations in transmission intensity in the different season and behavior of individuals to water contact. Thus, the difference in frequency and duration of water contact among individuals might also be a reason for the variable treatment effectiveness between those who were cured completely and those who were not cured post-treatment [29].

Another difference in effectiveness results might be also dead eggs were found with stool examinations, in those cured and might be considered as not cured, but this is rare phenomena. On the other hand, there was inability to detect eggs from fecal sample of numerous patients with low worm burden, while it was diagnosed using Kato’s technique [6]. This might be a reason for the overestimation of parasitological CR and the differences in effectiveness.

The effectiveness acquired by administering two dozes of 40 mg/kg PZQ with close interval of 3–6 weeks against schistosomiasis has been recommended as a way of advancing PZQ effectiveness [30, 31]. Immature worms (schistosomula) are mostly unmanageable to PZQ. Hence, administering a second dose of PZQ after number weeks ought to kill the worms as they get matured. Higher ERR and CR was reported in studies that used the administration of two doses of PZQ with close interval [31, 32]. Our effectiveness evaluation showed lower ERRS of (78.3%) than earlier reports that used two closely spaced doses of PZQ. The main reason might be also as our evaluation was based on single dose of PZQ.

One typical difference of the present study results and other previously reported results was that, in the present study, there was high CR (86.9%) but low ERR (78.3%) but in others the reverse was true. In our study, the possible reason might be due to pre-patent-infection and occurrence of a huge number of less susceptible juvenile schistosome worms during pre-treatment. The above possible reasons could also be supported by the increment in egg count (ERR of − 17.3%) from pre-treatment during the 4 weeks after treatment which decreased the total rate of egg reduction.

This result was concordant with study done in Wondo Genet, Southern Ethiopia (27). This indicated that in areas of intense transmission immature stage may predominate during treatment time and as praziquantel lucks effectiveness against the immature stage, during post-treatment the immature stage progress in to adult and still excrete ova of *S. mansoni*.

## Limitation of the study

The main limitation is the samples are taken from children that attend the school, and thus extrapolating the percentage infected to the general population is unjustified. On the other hand, this study is not clinical trial. Thus, it is difficult to test the effectiveness accurately. The study used single and only Kato–Katz slide for both the baseline and post-treatment for the *S. mansoni* egg count which lacks macroscopic stool investigation. This might underestimate the number of egg counted as light intensity of eggs might be missed.

## Conclusion

Overall, high prevalence of *S. mansoni* infection in Elementary school children was observed. In this study, praziquantel showed a CR of 86.9% and an overall ERR of 78.6% 4 weeks post-treatment. According to WHO standard, the efficacy (evaluated by CR and ERR) of this drug is in the normal range of (70–90%) to be used in treating schistosomiasis. It recommended both regional and national program managers, Tacharmachiho district health officers take appropriate control measures for the high prevalence of infection among school-aged children and mass drug administration should be started soon.

## Data Availability

Data is available upon request.

## Abbreviations

ANRSHB: Amhara National Regional State Health Bureau
CR: Cure rate
ED50: Effective dose
EPG: Eggs per gram
ERR: Egg reduction rate
GM: Geometric mean
GMEPG: Geometric mean egg per gram
IDs: Identifiers
MDA: Mass drug administration
PZQ: Praziquantel
SOP: Standard operating procedure
WHO: World Health Organization
